# Environmental Immersion’s Influence on Hedonics, Perceived Appropriateness, and Willingness to Pay in Alcoholic Beverages

**DOI:** 10.3390/foods8020042

**Published:** 2019-01-26

**Authors:** Benjamin Picket, Robin Dando

**Affiliations:** Department of Food Science, Cornell University, Ithaca, NY 14853, USA; bp299@cornell.edu

**Keywords:** taste, sensory evaluation, context, virtual reality, immersion, hedonics, alcoholic beverages

## Abstract

The eating experience is multimodal. As we consume a dish, we perceive much more than that which initially activates the senses, including influences from our surroundings. Foods sampled in experimental settings are largely evaluated within a sensory booth, an environment designed to be devoid of such external or non-standardized stimuli, so that participants can focus solely on the sample itself. In natural experiences, we rarely consume food in such isolation—context is actually key to many dining experiences and can have an integral role in how we perceive the foods we eat. Using virtual reality to artificially provide this context, we tested how the setting in which a beverage was consumed influenced perception of two different samples. Virtual environments were formed by processing custom-recorded 360 degree videos and overlaying audio, text, and sensory scales to simulate a typical sensory evaluation. Participants were asked to taste two alcoholic beverages, a beer and a sparkling wine, in two virtual contexts, a bar and a winery. The results indicated that participants’ willingness to pay for, and overall enjoyment of the sparkling wine increased when placed in the winery context, with no change between the two virtual contexts for the beer sample. This occurred without alteration of the samples’ sensory properties or the ability of panelists to identify the beverage they were drinking; however, perceived appropriateness of the samples for the setting was strongly influenced by the context in which they were sampled, suggesting that perceived appropriateness for a surrounding may play a role in the degree to which we enjoy a food. Results provide further proof that artificially-applied context, such as that provided by virtual reality, can further the sensory testing of foods.

## 1. Introduction

When a food is consumed, our taste buds only extract the degree to which the food is sweet, sour, bitter, umami, and salty, with proposed roles for the detection of a few more basic taste qualities such as fat, carbonation, or starchiness. However, the flavor of a dish does not derive from the basic taste characteristics of the dish alone; their role in the ultimate perception of a dish is part of a larger process, involving input from each of the senses, as well as input from experience, expectation, and our surroundings. Perhaps the most characterized of these are crossmodal interactions between taste and olfaction. A well-documented example of this concerns our enhanced ability to detect and identify tastes when exposed to congruent odors [1,2,3]. Evidence of the impact of vision on taste include sommeliers assessing white wine dyed with red food coloring as red [4], yellow or green food coloring influencing our perception of sour taste, and red resulting in the anticipation of something sweeter [5,6,7], possibly linked to expectation. Perhaps the most familiar visual element of food is the presentation of the dish. Plating is more than just visual stimulation, it influences the perceived flavor of the dish itself [8]. Dishes plated in an artistic manner are rated as tasting better than dishes containing the same ingredients plated in a simpler way [9]. 

Sound is also recognized as having a powerful impact on the taste characteristics of a food, influencing perception of the basic taste, texture, carbonation, freshness, and enjoyment of foods [10,11,12,13,14,15,16]. Interestingly, this effect may extend to the background noise of the setting in which the food is being consumed. Listening to jazz background music results in higher pleasantness ratings of chocolate, an “emotional” food, compared to bell peppers, a more neutral food [17]. Again, this may be linked to congruency (i.e., emotional music with emotional food) modulating pleasantness [18,19]. Background music in bars has also been shown to impact one’s ability to discern alcohol content, due to the fact that loud background music increases perceived sweetness (and, thus perhaps also perceived alcohol content) [20]. With classical music playing in the background, shoppers will often buy more expensive items [21]. Stores will sell more French wine if French music is playing, or sell more German wine if German music is playing [22]. Interestingly, the way in which food is consumed can also be altered by the background music, with panelists taking more bites per minute if there is background music of a fast tempo playing [23]. Not only do our senses play a role in the ultimate perception of a stimulus, but so too can the temperature of the food [24], our expectations [25], and even our own physiology [26,27,28].

Context, the environment in which a food is consumed, plays a vital role in food acceptance. Rozin & Tuorilla [29] go so far to imply that to ignore contextual influence on food choice and intake is to risk misinterpreting the meaning and significance of human food choice. At any point in time, one’s sensations are influenced both by events that occurred prior to consumption and those expected to occur post-consumption. By changing the decor of a restaurant from a neutral theme to a more Italian-themed setting, diners’ food selection significantly changes [30]; diners order more pasta dishes and dessert items. In addition, customers believe that the foods they were presented with were more authentically Italian when Italian-themed decorations were present. Diners have expectations of the type of food that a particular setting will deliver. One expects an expensive dinner location to have features distinguishing it from a cheap one with regard to ambience, formality, appropriateness of drinking alcohol, and so on [31]. As a result, changing the decor of a restaurant impacts the expectations of the consumer regarding the type of food they will be served, and how authentically that food will be prepared. These data are valuable for the restaurant industry, and for food and beverage corporations trying to understand the impact the environment can have on consumers’ experiences around their products. Importantly, the context in which a food product is being sold is often quite different from the environment in which it is consumed [32]. This could mean that a product accepted by a consumer in a taste-test at a supermarket can be rejected when the experience differs in the home environment. 

In recognition of this fact, sensory science has recently become interested in studying the influence of artificially applied context. Written scenarios given to a panelist are meant to personalize the consumption context for that participant. In these written scenario sensory tests, hedonic responses to the product can change significantly under this evoked context [33,34]. Coffee sample receives more favorable ratings in a simulated or evoked café [34,35]. Petit & Siefferman [36] found that ratings of acceptability for the same food were significantly higher when tested at home, and lowest in a hospital setting. Multiple studies agree that placing participants in differing contexts has a strong influence on the perceived acceptability of the food [37,38,39]. When testing a food, situations closer to the consumer’s typical consumption setting can gauge panelists’ reactions in a more representative setting and, consequently, the data collected are more ecologically valid. Clearly, any company looking to consumer-test a product should be cognizant of the fact that laboratory settings almost always induce lower acceptability ratings [39,40,41]. 

While at-home consumer tests and tests taking place outside the laboratory do indeed provide more ecological validity to sensory evaluation studies, studies that incorporate context in some capacity are not without their faults. Importantly, tests taking place outside the laboratory often lack a controlled setting—there are too many variables to control in the real world [36]. Therefore, researchers are forced to either conduct their studies in a more controllable lab setting or deploy their experiment in an ecologically valid setting that lacks this control. This is where emerging virtual reality (VR) technology may have a valuable role to play. The use of VR in sensory evaluation offers the ability to immerse a participant in a real-world setting while remaining in a controlled environment [42]. 

The goal of this study was to obtain insight into the extent to which context plays a role in sensory evaluation, preference, and willingness to pay in alcoholic beverages (consumed in a virtual bar vs a winery). We evaluated a complex multimodal environmental immersion using virtual reality to improve ecological validity. We hypothesized that altering the testing environment to better match the environment in which a consumer would encounter these products would improve hedonic response to the product, as well as perceived appropriateness and willingness to pay, and that congruency with context would make it easier for panelists to identify the samples. 

## 2. Materials and Methods

### 2.1. Virtual Environments

Environments were designed such that each sample could be experienced in both a congruent and an incongruent VR setting, and the experiences compared. The degree of difference between our setup and a standard sensory booth remains notable (i.e., presence of a VR helmet, restriction of vision, the wearing of headphones, and music during evaluation), however several previous studies compare real [43] and virtual sensory booths [44] to immersive testing environments. Videos were of two differing environments, a typical college bar (Loco Cantina, Ithaca, NY, USA) and a tasting room at a relatively expensive local winery (Hermann J. Wiemer Vineyard, Dundee, NY, USA), and were each approximately 3 min in duration. To create the two experimental contexts, 360 degree videos were recorded with a Samsung Gear VR 360 camera (Samsung Group, Seoul, Korea) and then stitched (a technique to digitally remove a joining line between the front and back cameras) using Samsung Gear 360 Action Director. Videos were cropped to an equal length with visual scales, on-screen selectable options, and audio instructions overlaid on the videos at identical positions and times using Adobe Premiere Pro CC 2017 (Adobe Systems, San Jose, CA, USA), with the files exported as H.264 mp4 files. Rock music was playing in the bar and classical music was playing in the winery; however, both were adjusted to a relatively quiet and equal volume, so that all verbal instructions would remain clear. Videos were viewed by the panelists on Samsung Gear VR headsets powered by Samsung Galaxy S6 phones. In addition to the aforementioned videos, an additional training video was prepared by our group to orient the panelists to the VR environment, the scales, and how to make ratings in the headset prior to the testing. During this video, panelists were asked to scale a variety of real and imagined stimuli on the generalized Labelled Magnitude Scale (gLMS) as well as the 9-point hedonic scale. Panelists rated samples by turning their heads to maneuver a cursor to the option or scale position they desired and pushing a button on the controller in front of them to take a screenshot of the image on the participant’s screen. The photo was then saved to the phone’s memory. All photos were uploaded in real time to Google Photos (Google Inc., San Francisco, CA, USA) so technicians alongside panelists could validate responses in real-time on a tablet connected to the same Google account. For a full standard operating procedure see Stelick et al. [44].

### 2.2. Participants 

A total of 59 panelists (60% female), verified as age 21 or above, recruited predominantly from the Cornell community, completed the study. All procedures were approved by the Cornell Institutional Review Board for Human Participants. Subjects were not informed of the objective of the experiment, and were not compensated for the session. Sessions lasted around 30 min in total, with panelists tested in a conference room equipped with two testing stations. Panelists ranged from 21 to 70 years old and were screened for any taste or hearing impairments in a self-reported survey before testing. Panelists were instructed prior to testing on the use of the scales across modalities and assessed to confirm they understood all instructions and could scale reliably. Participants were also screened for food allergies and epilepsy. 

### 2.3. Stimuli

**Figure 1:**
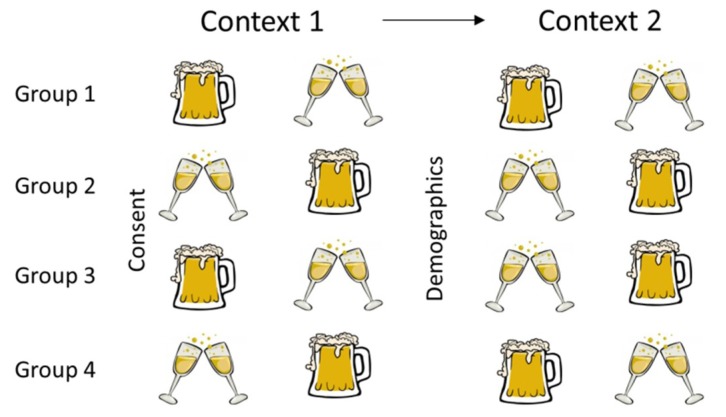
Diagram of the study’s set-up. Participants were placed in either the bar or winery, tasted one of each sample, changed context, and tasted each sample once more.

Two stimuli were prepared for use during the experimental trials: Veuve Du Vernay Brut (Veuve Du Vernay, Bordeaux, France) sparkling wine, and Miller High Life (Miller Brewing Company, Milwaukee, WI, USA), which is known as ”the champagne of beers”. For the purpose of clarity, the Veuve Du Vernay is referred to as champagne throughout the study, however, sparkling wine from Bordeaux is not strictly champagne; it is instead referred to as Cremant de Bordeaux. Samples were purchased from local vendors and were refrigerated and sealed when not in use. Fresh samples were opened each day of the study. The stimuli were selected based on the preliminary assessment that they differed enough in sensory properties to obtain discriminating responses, but were both alcoholic beverages, of similar sweetness, carbonation, and mouthfeel. Samples were poured immediately prior to serving to prevent loss of carbonation. 1 oz was served in a translucent 6 oz plastic cup. Stimuli were not assigned blinding codes as participants were already blinded due to the nature of the VR headset. Each sample was sequentially presented to the panelist by a technician, in pace with the video timing, and in a balanced order among the panelists. Samples were placed on a tactile plate in front of the panelist by the technician, whereby the panelist could feel where the cup would be on the plate when instructed a sample was ready to test. Panelists received a total of 4 samples, one of each sample in both virtual environments (see Figure 1). 

### 2.4. Experimental Design

Panelists were given an instruction packet about the headset, signed a consent form, then watched the training video. Participants were then placed in either the bar or winery context to begin testing, with the context switched halfway through the study. Both the order of drink presentation and contexts were counterbalanced per set (see Figure 1). 

At the halfway point of the study (after 2 samples had been consumed, and prior to the change in context), panelists removed the headset, cleansed their palates with water and unsalted crackers, and were asked a series of demographic questions, as well as how immersed they felt in the VR environment (from “not present at all” to completely present”). Panelists evaluated samples in a monadic sequential manner, first rating overall liking on a 9-point hedonic scale, then rating sample sweetness, bitterness, and carbonation on the gLMS, followed by willingness to pay (continuous line scale, from zero to $10) and appropriateness of the beverage relative to context for each sample (continuous line scale, from “not appropriate at all” (0) to “very appropriate” (100)). Finally, panelists were asked to identify the sample from a list of several options: beer, champagne, sparkling cider, seltzer, or ginger ale. 

### 2.5. Data Analysis 

Data were analyzed with linear mixed models for each dependent variable using IBM SPSS (IBM Corp., Armonk, NY, USA), and with paired t-tests using GraphPad Prism 5 (GraphPad Software, La Jolla, CA, USA). Terms of interest were tested for significance, and retained in the model to control for any additional variance they introduced if *p* < 0.1. In linear mixed models, panelist ID was used as a random effect, and panelist sex was retained as a fixed effect along with context, as this approached significance (*p* < 0.1) in several models. Pearson’s correlation analysis was also performed between rated degree of immersion vs differences in willingness to pay, appropriateness, or liking ratings between environments. Statistical significance was assumed at *p* < 0.05.

## 3. Results

**Figure 2:**
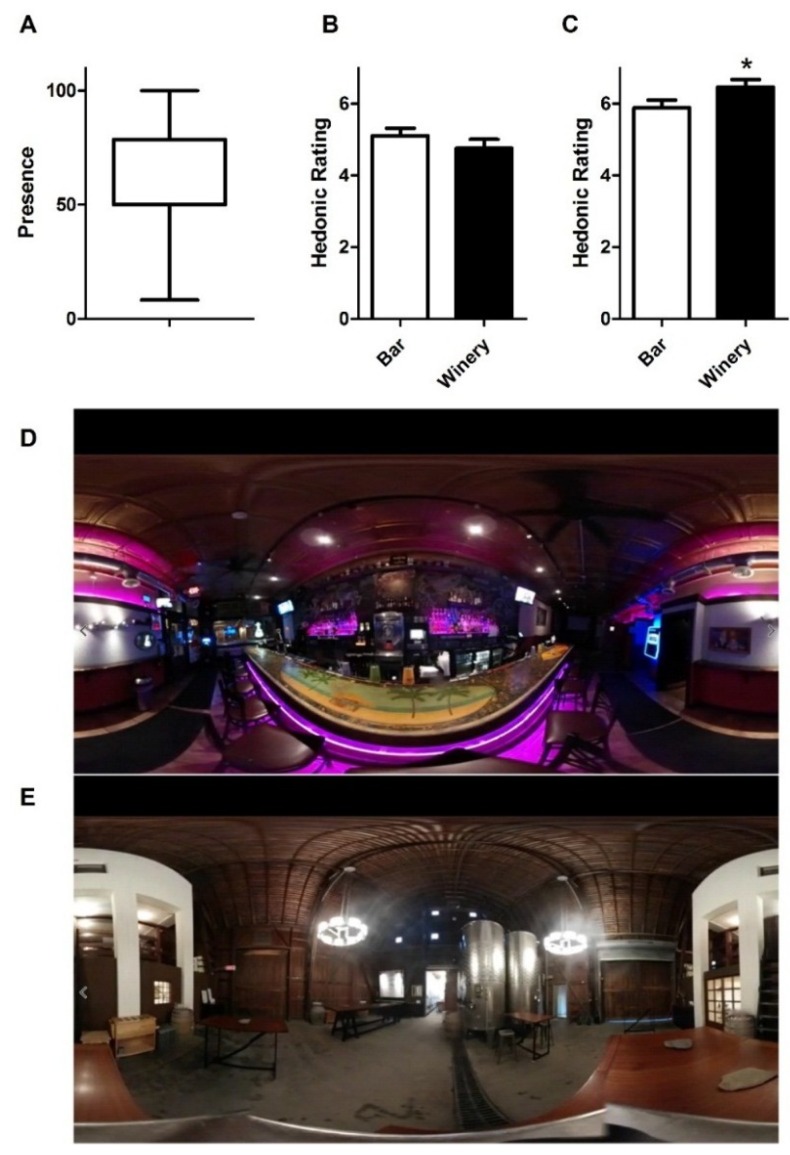
(**A**) Panelists’ feelings of immersion were rated from “not present at all” (0) to “completely present” (100). The box and whisker plot denotes mean of 54 and range (standard dev. = 22). (**B**) Liking for beer was higher, although not significantly, in the bar context (white bar) than the winery (black bar), plus standard error. Samples were rated on a 9-point hedonic scale, with labels indicating “dislike extremely” (1) to “neither like nor dislike” (5) to “like extremely” (9). (**C**) Panelists had higher hedonic ratings for champagne delivered in the winery context as compared to the bar; bars denote mean plus SEM. Asterisk denotes *p* < 0.05. (**D**) 360 degree virtual bar context. (**E**) 360 degree virtual winery context.

Results from the session revealed that 75% of consumers reported, at a minimum, a moderate degree of immersion within the system (Figure 2A). Student’s two-tailed, paired *t*-test showed that consumers displayed a significantly higher liking (*p* = 0.015, *t* = 2.50) for champagne than for beer while in the winery context (Figure 2C). Mean liking for beer was also slightly higher than for wine in the bar context, although not significantly (Figure 2B, *p* = 0.460, *t* = 0.7437). 

While hedonics varied with context, no statistical differences in the perceived intensity of sweetness, bitterness, or carbonation was found in either sample between the two contexts (Table 1; all *p* > 0.05). Additional factors (gender, scale usage, reported degree of immersion) were tested within the linear mixed model, with only gender influencing perceived intensity of bitterness from the samples (*p* = 0.008). Females perceived more bitterness from the samples (EM means: female = 18.8; male = 11.1), with a two-way interaction between gender and sample demonstrating that beer was the sample that female consumers found to be more bitter. 

**Figure 3:**
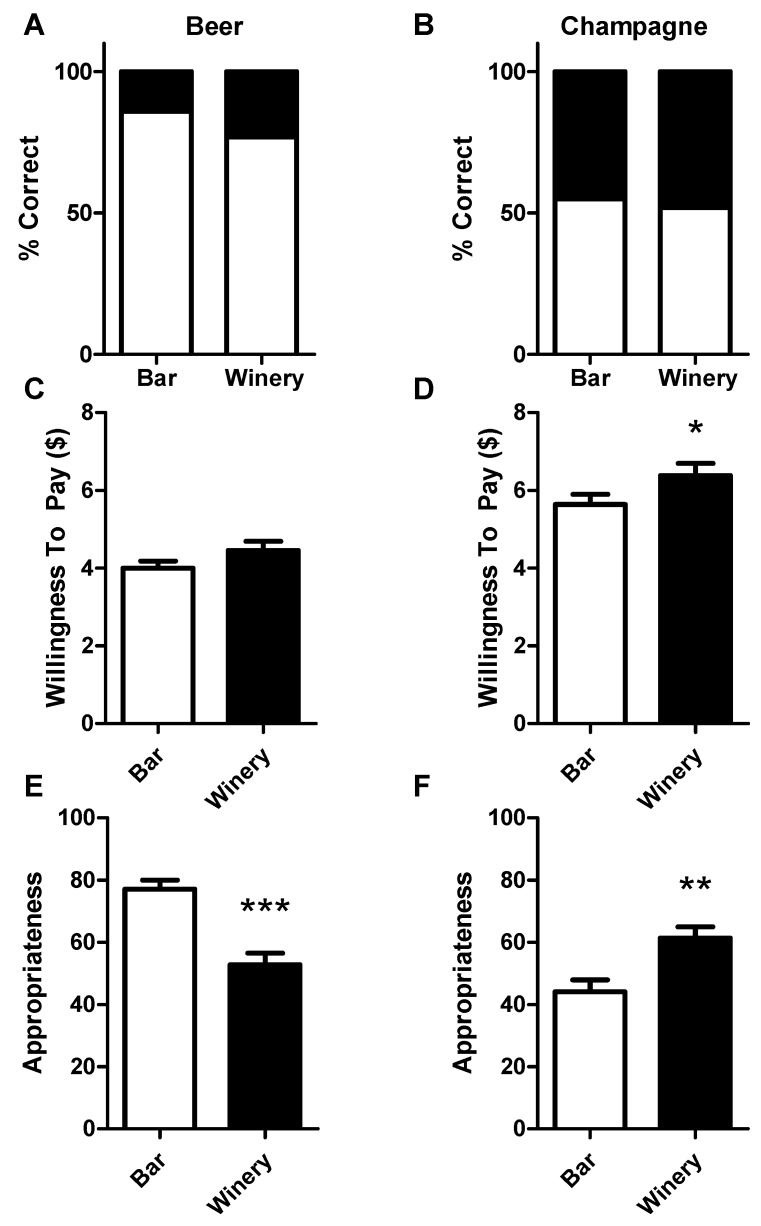
(**A**) Panelists’ identification of beer samples presented in the study (correct identifications in white, incorrect in black). (**B**) Panelists identified the champagne sample approximately as well in both the bar and winery contexts. (**C**) Panelists showed no difference in the amount they were willing to pay for beer in either context; bars denote mean plus SEM. (**D**) Panelists were willing to pay almost a dollar more for the champagne sample while in the winery than the bar context; bars denote mean plus SEM. (**E**) Beer appropriateness for context, from “not appropriate at all” (0) to “very appropriate” (100); bars denote mean plus SEM. (**F**) Champagne appropriateness for context; axis as E; bars denote mean plus SEM. Asterisks denote: * *p* < 0.05; ** *p* < 0.01; *** *p* < 0.001.

No difference in the ability to identify samples was found in either context (Figure 3A,B), However consumers’ willingness to pay for the samples was influenced by context. Panelists were willing to pay more for champagne in the winery context (Figure 3D; increased from an average of $5.64 to $6.38; *p* = 0.023, *t* = 2.333), though not for beer in the winery context (Figure 3C; *p* = 0.060, *t* = 1.921). The ratings of the appropriateness of the two environments for the sample in question was also tested. Both contexts, the winery and the bar, exerted a strong influence on the perceived appropriateness of the beverage that the panelists were sampling, with beer rated as being significantly more appropriate for the bar context (Figure 3E; *p* < 0.001, *t* = 4.379) and champagne, conversely, considered more appropriate in the winery context (Figure 3F; *p* = 0.005, *t* = 2.958). 

## 4. Discussion

In this report, we used 360 degree immersive visual and audial cues to create an artificial sense of presence within typical contextual settings of alcoholic beverage consumption. These contexts revealed stark differences in panelists’ perceptions of the samples’ appropriateness for each setting. In doing so, panelists’ liking of champagne increased in a virtual winery setting as compared to a bar, while contexts did not change the products’ sensory properties, or the panelists’ abilities to identify samples, suggesting that perceived appropriateness for a setting may alter enjoyment of a sample. That no difference was seen in liking between contexts for the beer sample could be a reflection of lower liking scores in general for the beer (means 5.1 and 4.8, close to neutral in both contexts); a weaker link between beer and the bar environment (not all bar patrons consume beer while there, which is in turn a broad category); or, considering the evident trend, could imply that a larger sample size would be needed to see differences, in turn implying that such effects are subtle. 

A concept is formed from both the virtual environment’s aspect (visual and audial), and any learned social relevance of the context. The sensory experience of a food is influenced by the physicochemical properties of the sample, as well as from input from this context (though not observed in this experiment) [44]. Identification of the sample may also possibly be influenced by context (though again, not in our experiment), but seems largely dependent on a panelist’s sensory experience of the sample. An alternative model we also proffer may be that liking and willingness to pay are directly influenced by the sample context; in a more elegant environment, we expect to pay more for the same product, and may even enjoy it more due to the alluring environs.

Our findings suggest that the presence of a contextual cue congruent with expectations of the setting that a beverage is typically consumed within were reinforced during the evaluation. These findings are in agreement with results found in Bangcuyo et al. [45] where participants most enjoyed coffee samples in a simulated coffeehouse setting. Additionally, the emotions elicited by a particular context may also bleed over into the individual judgments participants make regarding a product’s hedonic properties [46].

Interestingly, although we previously found that different contexts evoked alterations in a product’s sensory characteristics with a VR approach [44], this was not evident in our results. Across both contexts, ratings for sweetness, bitterness, and carbonation remained unaltered. This was most likely due to the deliberate selection in Stelick et al. [44] of a setting predominantly associated with one of the attributes being measured. Conversely, we would reason that neither sweetness, bitterness, or carbonation are more readily associated with a bar versus a winery, or vice versa.

We anticipated that willingness to pay would increase in the more “classy” winery context, consistent with findings from Liu et al. [47] that participants were willing to pay more for the same “all natural” food products when they were delivered in the context of a farmers’ market as compared to a supermarket. Similarly, the winery setting in our study induced panelists to report a willingness to pay more for the champagne in the winery. Conversely, the beer sample remained valued at a similar price point, likely due to the incongruence between sample and context, with panelists not freely associating beer with the winery context. Studies have previously demonstrated that price can influence the perceived quality or taste of a food or beverage [48]. Indeed, in our study, the Pearson’s correlation coefficient between liking and willingness to pay was 0.520 (*p* < 0.001). Consumers typically associate more expensively priced products as being higher in quality, and this expectation directly influences sensory judgments. Even sommeliers, who are classically trained in wine, assume that higher priced wines are better and, as such, may alter their hedonic ratings accordingly [48]. Consumers’ impressions that the beer was more appropriate for the bar and that the champagne was more appropriate for the winery suggests that there are indeed schemas that guide our understanding of where a food or beverage should be consumed. Certain settings better fit prototypes that one develops over the course of one’s life. This is likely why participants found the beer more appropriate in the bar context and champagne more appropriate in the winery. That the champagne was more liked in the winery may have been a reflection of this increased appropriateness, but could also have been a direct reflection of liking induced by the environment itself; however, that the beer was unchanged would suggest the former. While our hypotheses regarding willingness to pay, appropriateness, and liking were corroborated by the data we gathered, it seemed that context did not have as much of an impact on identification of the sample as we predicted. The associations between a particular context and the beverages we are accustomed to drinking in that setting may exert strong enough top-down influences to override the information provided by our senses. Beverages sampled from containers that were incongruent with one’s expectations (i.e., beer presented in a coffee cup) were consistently rated lower than the same beverage presented in a container that was congruent with expectation [49]. 

75% of our participants felt at least moderately immersed in the VR environment. That a minority did not feel suitably immersed indicates that further improvement on the VR system is possible. The most prominent issues were that the samples remained invisible to the panelist while in the headset and that there was a lack of a personal avatar while in the environment (which may have subtracted from the sense of presence notably). In further analysis, we found no correlation between those reporting a high degree of immersion within the environments, and disparity between measures reported between contexts (liking, willingness to pay, or appropriateness; all *p* > 0.05). 

Limitations to the project include the fact that participants were visually blinded from the samples they were testing and were thus devoid of visual information regarding the beverage. In this sense, the study was not able to quite replicate the way in which the average consumer would interact with the product. In our design, we did not make a direct comparison to a sensory booth, however, we note that other research has shown notable differences between sensory experience in booths versus more immersive experiences [45,43].

## 5. Conclusions

This study, alongside other emerging research in VR, demonstrates that real-world digital renderings can alter the appreciation of foods and beverages, at least in some settings, in a manner relevant to researchers and brands alike. Evidence from this study and others highlights the potential for the expansion of traditional sensory testing to include immersive technology that may provide data more relevant to the actual consumer experience, adding ecological validity to testing via the modeling of real food environments. In this study, we showed that altering visual and audial surroundings, in the case of the more “classy” winery context, influenced a participant’s willingness to pay, liking, and feelings of appropriateness of a congruent product from an entirely virtual environment. Results show promise that, with further study, technology such as this may be utilized to offer additional versatility to consumer sensory testing, and may be of interest in the study of factors such as dining ambience, social context, and eating behaviors in specialized environments such as hospitals, airplanes, or schools. 

## Figures and Tables

**Table 1:** Ratings of sweetness, bitterness, and carbonation of beverages in two virtual environments.

		Bar		Winery	
		Mean	St Dev	Mean	St Dev
Sweetness	Beer	10.4	7.7	11.0	8.8
	Champagne	20.9	13.7	20.8	14.2
Bitterness	Beer	17.9	11.8	18.6	15.5
	Champagne	13.6	10.1	12.6	10.5
Carbonation	Beer	18.0	10.8	15.2	9.6
	Champagne	25.1	14.7	26.2	14.3

Panelists rated the sweetness, bitterness, and carbonation of the beer and wine samples on the gLMS as similar in either context (all *p* > 0.05). Samples were rated on the gLMS, with labels indicating “barely detectable” (1), “weak” (6), “moderate” (17), “strong” (34.7), “very strong” (52.5), and “strongest imaginable sensation of any kind” (100).
